# Advancing modified biochar for sustainable agriculture: a comprehensive review on characterization, analysis, and soil performance

**DOI:** 10.1007/s42773-024-00397-0

**Published:** 2025-01-03

**Authors:** Ali Fakhar, Snowie Jane C. Galgo, Ronley C. Canatoy, Mazhar Rafique, Rubab Sarfraz, Aitazaz Ahsan Farooque, Muhammad Israr Khan

**Affiliations:** 1 Department of Biological and Chemical Engineering, Aarhus University, Aarhus, Denmark; 2 Institute of Agriculture & Applied Life Science, Gyeongsang National University, Jinju, 52828 Republic of Korea; 3 Department of Soil Science, College of Agriculture, Central Mindanao University, 8710 Maramag, Philippines; 4 Department of Soil and Climate Sciences, The University of Haripur, Haripur, Khyber Pakhtunkhwa Pakistan; 5 Canadian Centre for Climate Change and Adaptation, University of Prince Edward Island, St Peters Bay, PE Canada; 6 Faculty of Sustainable Design Engineering, University of Prince Edward Island, Charlottetown, PE C1A4P3 Canada; 7 College of Agriculture, Sultan Kudarat State University, Lutayan Campus, 9803 Philippines

**Keywords:** Pristine biochar, Soil organic carbon, Functionalized biochar, Material saturation, Complexation, Manure-derived biochar

## Abstract

Biochar is a carbon-rich material produced through the pyrolysis of various feedstocks. It can be further modified to enhance its properties and is referred to as modified biochar (MB). The research interest in MB application in soil has been on the surge over the past decade. However, the potential benefits of MB are considerable, and its efficiency can be subject to various influencing factors. For instance, unknown physicochemical characteristics, outdated analytical techniques, and a limited understanding of soil factors that could impact its effectiveness after application. This paper reviewed the recent literature pertaining to MB and its evolved physicochemical characteristics to provide a comprehensive understanding beyond synthesis techniques. These include surface area, porosity, alkalinity, pH, elemental composition, and functional groups. Furthermore, it explored innovative analytical methods for characterizing these properties and evaluating their effectiveness in soil applications. In addition to exploring the potential benefits and limitations of utilizing MB as a soil amendment, this article delved into the soil factors that influence its efficacy, along with the latest research findings and advancements in MB technology. Overall, this study will facilitate the synthesis of current knowledge and the identification of gaps in our understanding of MB.

**Graphical Abstract:**

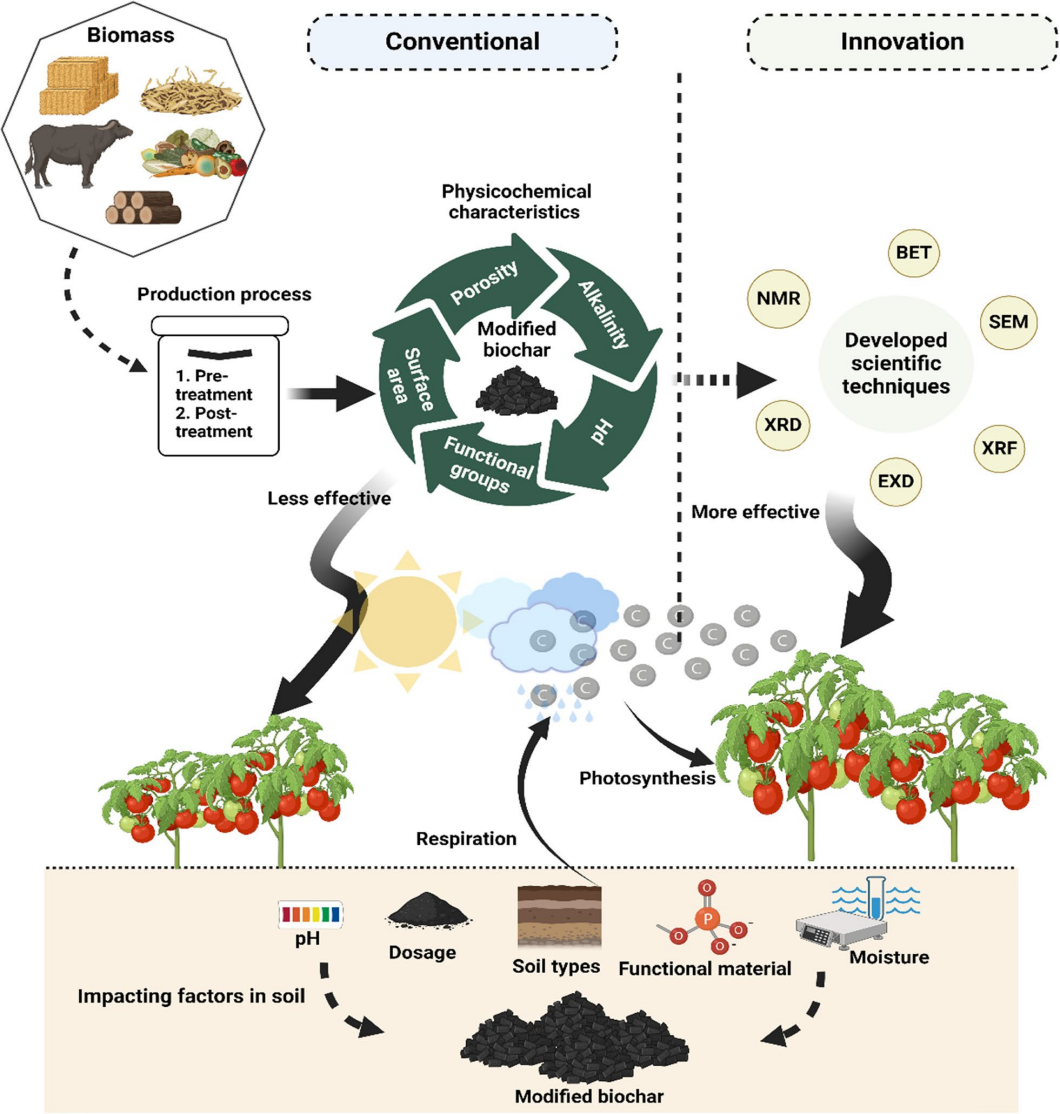

## Highlights

1. The application of modified biochar significantly increased over the past decade.
2. Modified biochar demonstrated promising properties for specific applications, particularly in soil.
3. However, outdated analytical techniques and limited understanding of soil factors could affect its efficiency.
4. The potential benefits, along with limitations in commercialization and future approaches, were highlighted.

## Introduction

Biochar is a carbon-rich material produced through pyrolysis of different feedstocks, such as wood, crop residues, or manure, in the absence of oxygen, typically containing around 85% more carbon than the original biomass (Ghani et al. 2013; Tsai and Liu 2016).

The modification of feedstocks or pristine biochar can be classified into three different types: activated biochar (AB), engineered biochar (EB), and functionalized biochar, based on the methods used to alter the biochar. AB is produced by exposing biochar to high temperatures in the presence of certain gases (such as CO_2_ or steam), which enhance its physicochemical properties (Braghiroli et al. 2018), while EB is modified by changing the feedstock or adding compounds like metal ions (MgCl_2_ and ZnCl_2_) (Monga et al. 2022). Biochar modified by adding functional groups to enhance its chemical properties is referred to as functionalized biochar (Qu et al. 2022). However, the term B generally distinguishes between pristine and altered biochar.

The studies regarding the use of MB in soil have shown a significant increase in recent years owing to numerous benefits, such as increasing soil fertility (Sultan et al. 2020), improving water retention, reducing nutrient leaching (Dvořáčková et al. 2021), climate change mitigation (Lehmann et al. 2021), and carbon sequestration (Lorenz & Lal 2014). The efficacy of biochar in soil can be disturbed by various factors, such as climate, application, and soil type. Additionally, the feedback from biochar application may not be consistent across all soils, as they were highly dependent on the physicochemical characteristics of the biochar (Panwar & Pawar 2020).

**Fig. 1:**
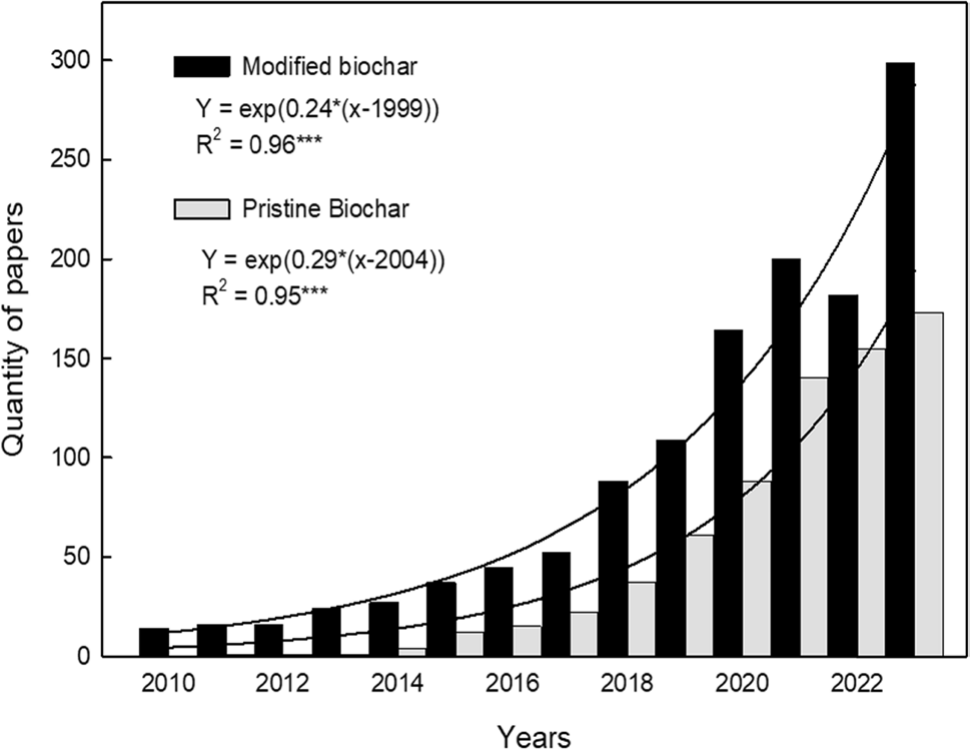
The number of published articles per year for a period from 2010 to 2023 retrieved using keywords “activated biochar,” “functionalized biochar,” “engineered biochar,” and “pristine biochar.” MB: modified biochar

**Fig. 2:**
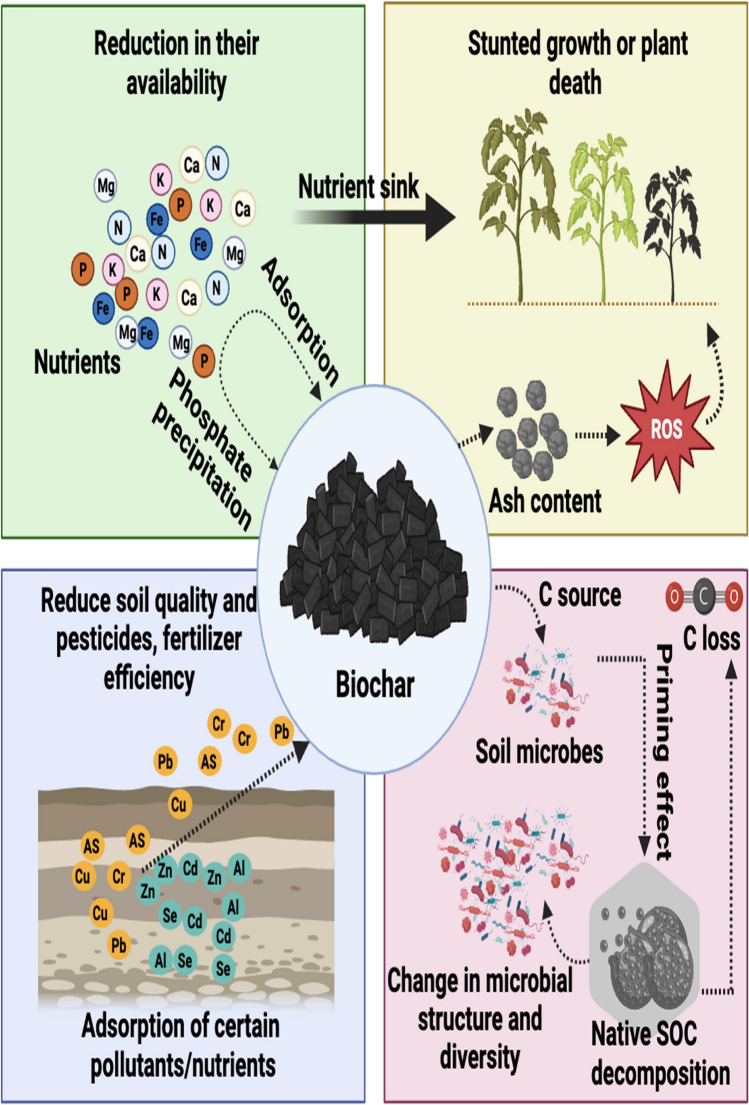
Fate and limitation of biochar in agricultural soils. (For interpretation of the color references in this figure legend, the reader is referred to the Web version of this article)

**Fig. 3:**
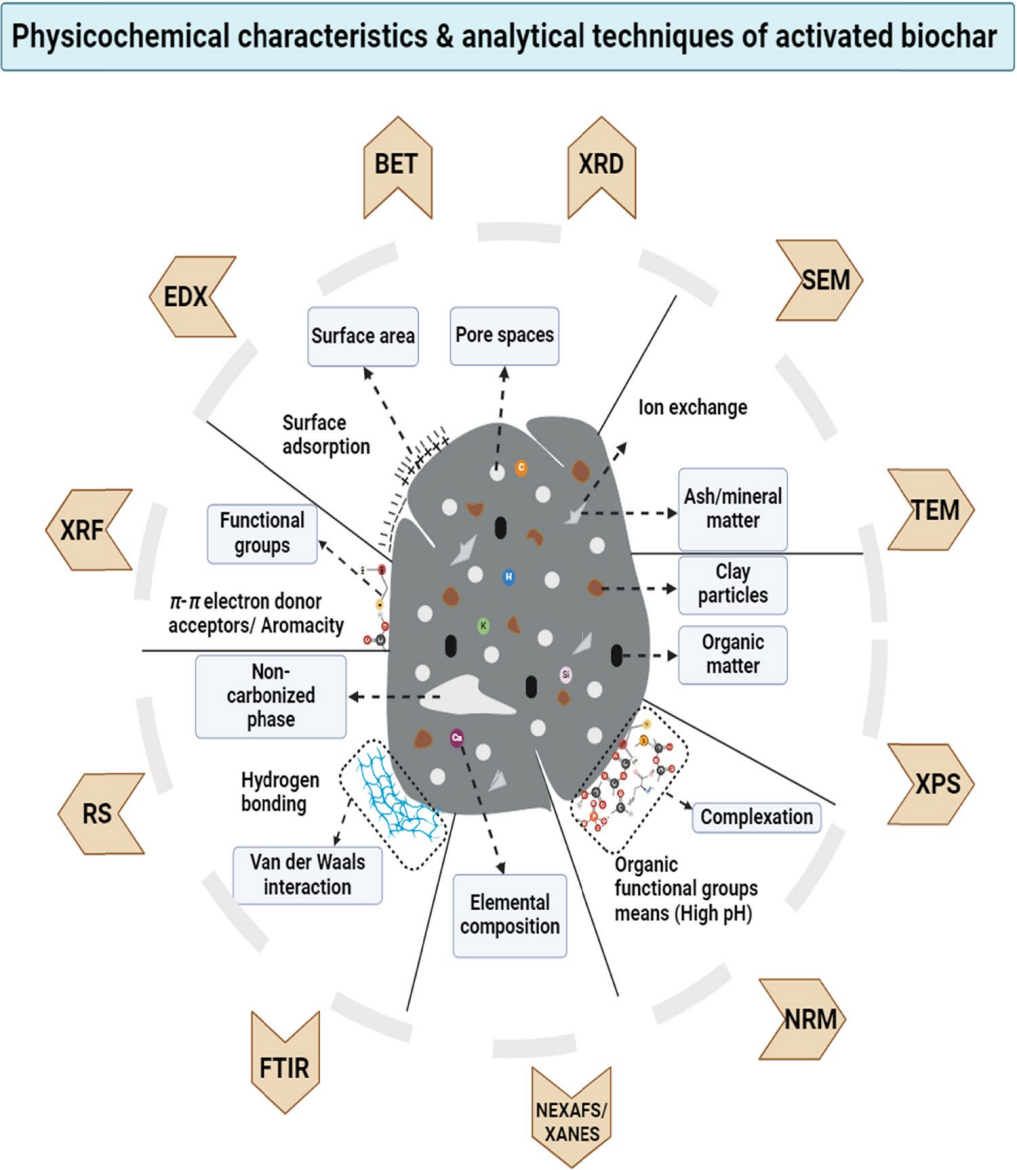
Physicochemical characteristics and analytical techniques of modified biochar. (For interpretation of the color references in this figure legend, the reader is referred to the Web version of this article)

**Fig. 4:**
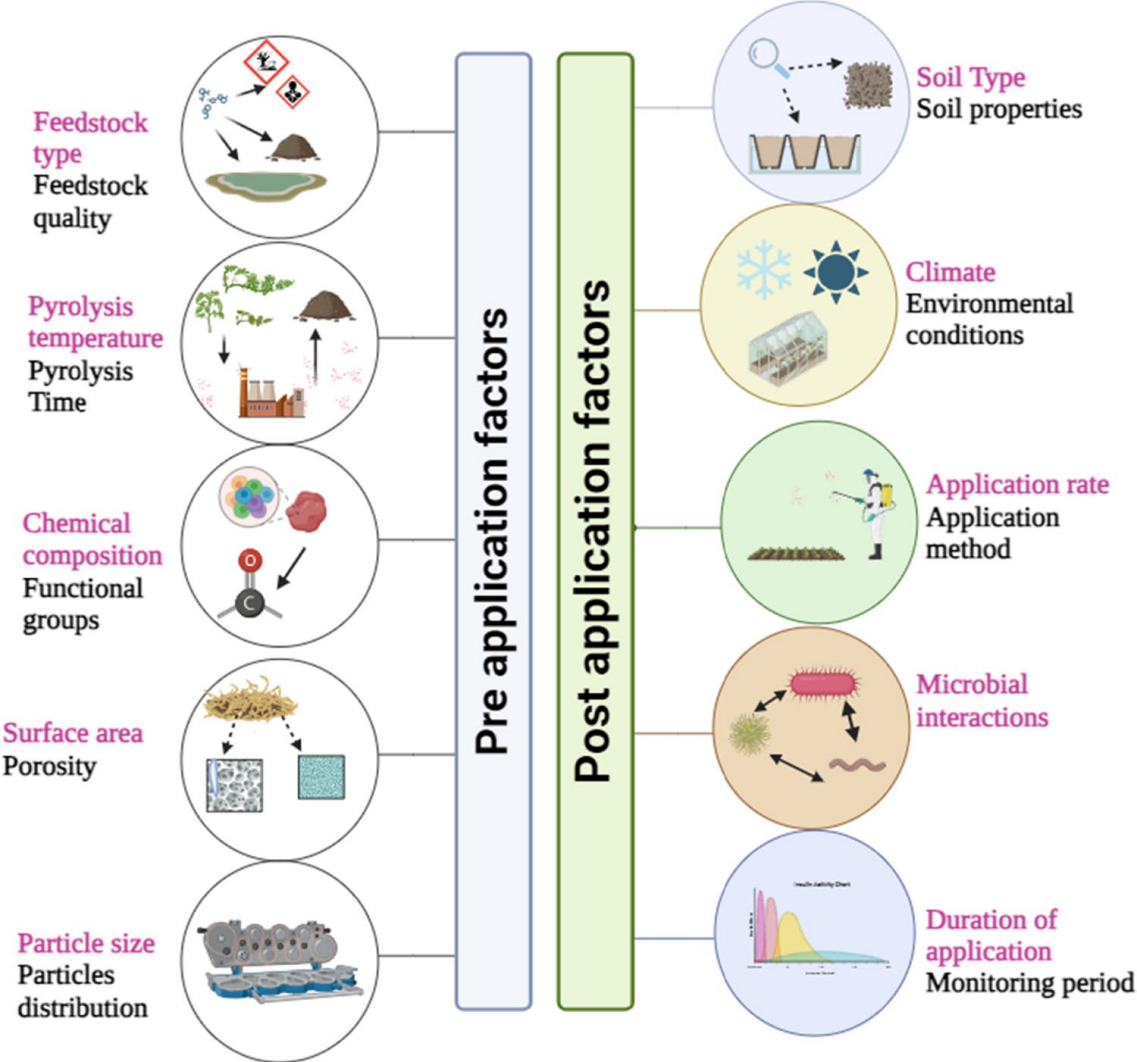
Factors impacting the efficacy of modified biochar following soil applications. (For color interpretation, the reader is referred to the Web version of this article)

**Fig. 5:**
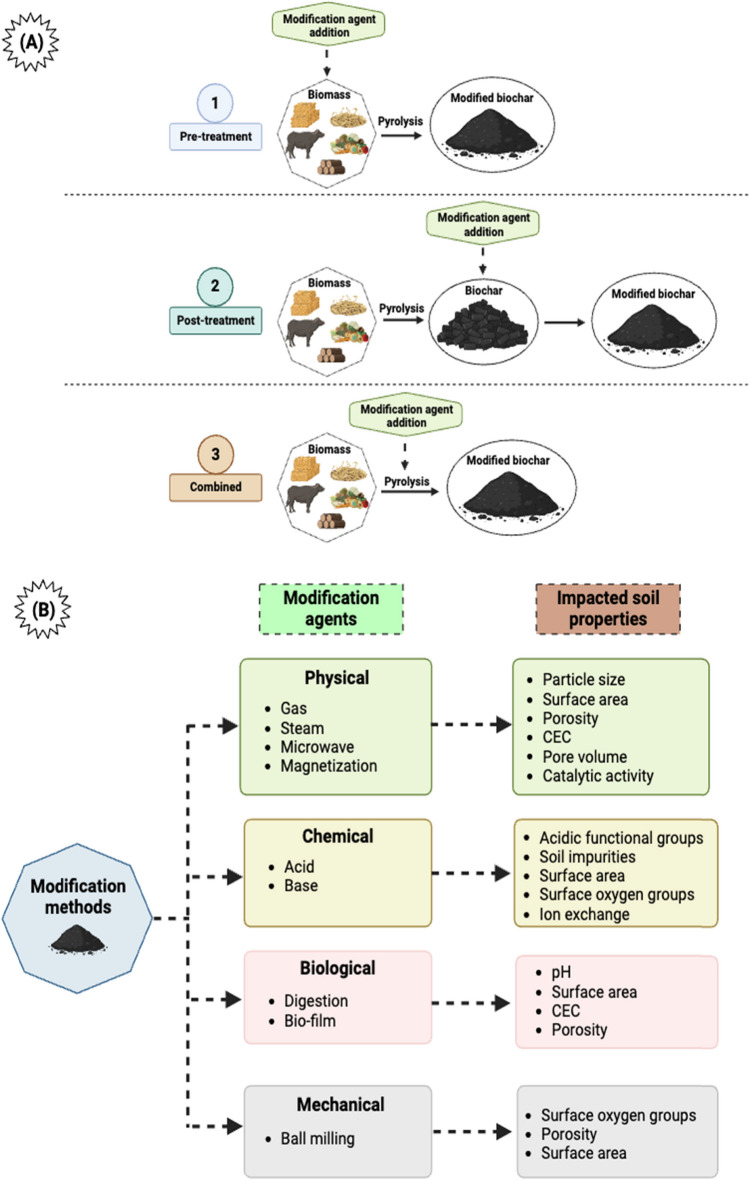
Factors impacting the efficacy of modified biochar following soil applications. (For color interpretation, the reader is referred to the Web version of this article)

Given the limitations mentioned earlier associated with the utilization of pristine biochar in soil, there is a notable shift toward the adoption of MB (Fig. 1). Thus, by adjusting the physicochemical properties of biochar, it becomes feasible to amplify its beneficial effects while concurrently mitigating any adverse impacts on plant growth and soil health (Lu 2015; Tan et al. 2021). However, before choosing any modification technique, it is imperative to have a comprehensive understanding of the various physicochemical properties of biochar that have been discovered and subjected to modification, as well as their respective advantages and limitations. Thereby, this review article provides an in-depth discussion of recent advancements in the understanding of physicochemical characteristics of MB, including surface area, porosity, alkalinity, pH, elemental composition, and functional groups (2010–2022). Additionally, it also highlights the factors that can influence the efficacy of MB in soil (Figs. 2, 3, 4, 5).

## Fate and limitations of biochar in agricultural soils

Several researchers have reported numerous findings regarding the fate and beneficial aspects of biochar. Apropos of soil physical properties, biochar amendment has been shown to improve soil pore structures and aggregate stability, which apparently upgrades soil water holding capacity. However, soil texture contributes a critical role in acquiring such benefits; for instance, biochar amendment is more noticeable in sandy soils than in clay soils. Hence, the positive feedback of biochar is not similar for all soil types (Blanco-Canqui 2017). Similarly, depending on pH and EC, different soil types react distinctively to the ash content delivered by biochar. The high levels of ash content can pose detrimental impacts on plants and may produce reactive oxygen species in soil (Das et al. 2020).

Similarly, the agronomical traits of plants can also be significantly improved by biochar application through upgrading nutrient cycling and soil environment (Alvarez-Campos et al. 2018). However, such positive effects may vary depending on the plant species and the targeted part of the plant because different plant species have distinct nutrient requirements, and biochar may not necessarily provide all the required nutrients. Biochar may also affect soil environmental conditions and the microorganism's diversity by disturbing the decomposition of organic matter, also known as the priming effect (Huang and Gu 2019). The impact may arise from changes in microbial communities induced by biochar, potentially resulting in either heightened or diminished decomposition of native soil organic carbon (SOC), consequently influencing its stability (Fig. 2). For example, microorganisms, specifically fungi species such as *Ascomycota* and *Basidiomycota*, are more effective at breaking down biochar and releasing nutrients for plant uptake, while others may be less efficient (Zhu et al. 2017). Similarly, Dai et al. (2018) found a significant decrease (11%) in the relative abundance of *Basidiomycota* by easily mineralizable C from biochar. The aforementioned limitations can be managed by modifying biochar to enhance specific chemical properties such as pH, cation exchange capacity (CEC), surface area, functional groups, and nutrient content that are beneficial for the targeted plants or specific plant parts. For example, the modifying process introduces new substances such as nitrogen, phosphorus, and potassium, which can minimize the adverse effects on SOM (soil organic matter) decomposition and soil microbial communities by interrupting C:N:P ratios (Naylor et al. 2022). Additionally, increasing the pH and CEC of biochar by activation and/or engineering methods can enhance its ability to retain nutrients and prevent leaching (Yuan et al. 2019).

Previously, Xu et al. (2016) reported that the combined application of biochar and phosphorus fertilizer in saline-sodic soil depicted a considerable decline in available P levels by accelerating phosphate precipitation/sorption processes owing to its high surface area and adsorption capacity (Fig. 2). Hence, it can act as a sink rather than a P source for plants. Biochar modification by altering its surface area, pore size distribution, and functional groups, can increase or decrease its adsorption capacity for specific nutrients and help to regulate its selectivity for specific soil nutrients while reducing its affinity for others (Jiang et al. 2018; Tran et al. 2022).

Previous studies enable us to refer that the capacity of biochar to adsorb essential nutrients such as nitrogen (N) or iron (Fe), sometimes surpasses the limits that are conducive to plant growth (Dai et al. 2020). If biochar adsorbs nutrients exorbitantly, it can lead to a reduction in their availability for plants, which can result in stunted growth or even plant death (Carter et al. 2013). Therefore, it is essential to carefully manage certain physicochemical characteristics to ensure that it does not have a negative impact on plant growth by competing with the plants for these essential nutrients. This can be achieved by using MB that has been specifically tailored to the needs of the soil and the plants being grown (Kavitha et al. 2018).

Furthermore, the adsorption abilities of biochar can be selective and biased towards certain pollutants, which can affect the effectiveness of pesticides, the concentration of heavy metals, and even plant defense mechanisms (Tian et al., 2021). However, researchers are exploring ways to adjust the surface chemistry of biochar to improve the targeted removal of specific pollutants while minimizing any negative impacts on plant growth and defense (Nkoh et al., 2022).

## Physicochemical characteristics of modified biochar

The physicochemical characteristics of MB (Table 1) vary based on various alteration techniques, pyrolysis temperature, and the kind of feedstocks and modifying compounds (Table 2). This section discusses how different modification methods and agents improve the physical and chemical characteristics of MB (Liu et al. 2021).

i. Surface Area and Pore spaces

**Table 1:** Overview of essential characteristics of biochar

Property	Definition	Reference
Anion exchange capacity (AEC)	The potential of biochar to adsorb anions	(Li et al. 2019; Tan et al. 2016; Weber & Quicker 2018; Xie et al. 2015)
Ash content	These residues are non-combustible byproducts resulting from pyrolysis, originating from mineral and inorganic constituents of biochar	
Cation exchange capacity (CEC)	The potential of biochar to adsorb cations	
Density	Density refers to the mass of a substance divided by its volume, factoring in any spaces between particles. A lower density indicates a lighter weight per unit volume	
Electric conductivity	It signifies the conductivity of a material, indicating its capacity to conduct electric current	
Elemental composition	It represents the mole ratios of oxygen (O), carbon (C), hydrogen (H), nitrogen (N), and sulfur (S). Typically, the ratios of O/C and H/C moles are used as indicators of the degree of carbonization, where low ratios often indicate a higher stability of biochar	
Fixed carbon content	The extent of carbonization can be inferred from the fixed carbon content, which is derived by subtracting the percentages of moisture, volatile matter, and ash from a given biochar sample. This calculation is expressed by the formula: FC (%) = [100 − (VM + Ash)]	
Heating value	A metric denoting the utmost thermal energy accessible from complete combustion, often referred to as energy content, is defined as the heat produced per unit mass or per unit volume	
Hydrophobicity	The attraction or affinity of biochar toward the water	
Mass yield	An indicator of biochar production efficiency, denoting the ratio of the mass of pyrolyzed products to the mass of raw biomass	
pH-value	An indicator of the alkalinity or acidity of biochar, expressed as pH, which is calculated using the formula pH = − log[H +]	
Pore volume and pore size distribution	The cumulative volume of pores and voids within biochar defines its pore space. The distribution of pore sizes signifies the proportional occurrence of each pore size within the structure of biochar	
Porosity	The ratio of the volumes of voids or pore space within a substance divided by the total volume of that substance is known as the porosity	
Specific surface area (SSA)	The SSA of a substance, calculated as the total surface area per unit mass, serves as an indicator of both adsorption capacity and water retention ability in biochar	
Stability	The percentage of original carbon content that remains following both abiotic and biotic degradation signifies the recalcitrance of the material in specific applications, such as carbon sequestration, under different conditions and time frames	
Surface functional group	The functional groups found on the surface of biochar, such as carboxylic (-COOH), hydroxyl (-OH), amine, amide, and lactonic groups, enhance its sorption properties. These groups indicate the biochar's capacity to adsorb organic compounds and pollutants effectively, as well as its catalytic performance The functional groups present on the surface of biochar, including hydroxyl (-OH), carboxylic (-COOH), amine, lactonic, and amide groups, play a crucial role in enhancing their sorption properties. These groups are indicative of the biochar's ability to effectively adsorb organic compounds and contaminations, as well as its catalytic performance	
Water holding capacity	The capacity of biochar to absorb and retain water

**Table 2:** Overview of specific physicochemical characteristics of modified biochar used as a soil amendment

Raw material	Feedstock	Activation	Pyrolysis Temperature (°C)	Modification Treatment	SA (m^2^ g^−1^)	pH	CEC (cmolc kg^−1^)	C	H	O	N	Reference
Plant	Water hyacinth	O	450	Post-treatment	32.48	10.3	3.53	46.7%	–	–	2.53%	(Mosa et al. 2018)
		Fe			155.91	9.4	6.35	50.3%	–	–	3.57%	
		Mn			34.34	9.4	3.73	47.6%	–	–	3.05%	
		Zn			95.65	8.8	6.04	50.4%	–	–	3.14%	
		Cu			62.11	9.4	3.93	49.1%	–	–	3.46%	
	Pine chips	HCl	400	Post-treatment	-	2.5	17.4	608 g kg^−1^	–	–	1,372 µg g^−1^	(Doydora et al. 2011)
	Peanut hull				-	2.5	15.7	625 g kg^−1^	–	–	1,8 µg g^−1^	
	Rice straw	FeOS	300	Post-treatment	37.4	-	-	37.4%	3.9%	0.8%	34.6%	(Wu et al. 2018)
		FeCl_3_			40.9	-	-	40.9%	4.7%	0.96%	30.8%	
		Fe			38.5	-	-	38.5%	3.4%	0.76%	23.6%	
		Fe^3+ (^1%)	500	Post-treatment	23.3	9.9	-	46.4%	2.8%	16.2%	2.23%	(Zhang et al. 2019)
		Fe^3+ (^5%)			26.4	5.7	-	43.8%	2.7%	15.8%	2.27%	
		Fe^3+ (^10%)			7.3	3.1	-	3.25%	2.5%	15.9%	1.72%	
		FeCl_3_ (1%)			14.6	10.2	-	45.5%	2.0%	11.2%	2.23%	
		FeCl_3_ (5%)			5.9	3.4	-	44.2%	2.1%	12.0%	2.32%	
		FeCl_3_ (10%)			5.7	3.1	-	44.1%	2.0%	13.8%	2.40%	
	Corn stem	Fe–Mn	600	Post-treatment	60.67	-	-	72.6%	2.2%	4.7%	1.2%	(Lin et al. 2019a, b, c)
	*Platanus orientalis* L	Fe	650	Post-treatment	74.5	4.4	-	59.9%	0.9%	–	2.2%	(Yang et al. 2022)
	Corn straws	FeCl_3_	600	Pre-treatment	4.1	2.5	-	38.0%	1.4%	27.7%	1.0%	(Fan et al. 2018)
	Rice hull	NaOH	450	Post-treatment	396	6.5	3.2	77.9%	3.4%	15.7%	1.7%	(Wang et al. 2021)
	Cracking crop straws	Commercial	500	-	22.9	8.3	–	414.7 g Kg^−1^	–	–	14.36 g Kg^−1^	(Hua et al. 2021)
	Rice straw	Thiol	500	Post-treatment	0.34	2.3	–	43.7%	0.6%	–	–	(Fan et al. 2020)
	Wheat straw	Mg	600	Post-treatment	292.5	–	–	54.5%	2.3%	15.4%	0.5%	(Zheng et al. 2020)
		Al			169.6	–	–	46.8%	3.1%	16.9%	0.3%	
		Mg–Al			268.5	–	–	43.0%	2.0%	12.9%	0.4%	
		Microbial	600	Pre-treatment	3.77	9.7	–	48.5%	5.8%	–	0.6%	(Muhammad et al. 2021)
	Corn straw	KMnO_4_	600	Post-treatment	3.18	10.7	–	73.0%	0.7%	10.9%	0.3%	(Yu et al. 2015)
	Rice husks	FeCl_3_	600	Pre-treatment	-	7.8	–	34.5%	1.0%	11.3%	0.3%	(Sun et al. 2021)
	*Platanus orientalis* L	FeCl_3_	650	Post-treatment	74.5	10.6		59.9	2.2		0.9	(Pan et al. 2021; X. Yang et al. 2021a, b)
	Carrot pulp	Thiourea CH_4_N_2_S	550	Post-treatment	–	9.1	59.18	47.2%	4.1%	23.2%	8.9%	(Gholami & Rahimi 2021; Gholami et al. 2020)
	Cotton straws	H_3_PO_4_	500	Pre-treatment	–	–	–	67.8%	4.1%	14.3%	0.11%	(Rizwan et al. 2020)
		NaOH			–	–	–	68.2%	3.9%	6.5%	0.18%	
	Corn straw	Fe (NO₃)₃	600	Post-treatment	207	9.8	1.0	68.0%	18%	–	2.4%	(Chang et al. 2021; Lin et al. 2019a, b, c)
Animal	Poultry manure	Chitosan	450	Post-treatment	3.7	10.4	–	11.3%	1.2%	–	–	(Mandal et al. 2017)
	Sheep manure				5.2	10.4	–	88.6%	1.0%	–	–	
	Pig carcass	FeCl_3_	650	Post-treatment	18.4	10.6		30.8	1.3		2.1	(Pan et al. 2021; X. Yang et al. 2021a, b)

*CEC* Cation exchange capacity, *SA* Surface area

The specific surface area (SSA) and pore structure of biochar play a significant role in adsorption and regulating the nature of biochar as hydrophilic or hydrophobic (Tan et al. 2021). The general purpose of modification in pristine biochar is to expand its surface area, which ultimately modifies its functional groups and enhances its magnetic performance and catalytic capacity (Wang and Wang 2019). Many studies showed that pyrolysis temperature and functionalization with innovative materials can enhance the SSA and porous structure of biochar (Leng et al. 2021; Tan et al. 2021; Zhou et al. 2020). Rong et al. (2019) observed a significant surge in surface area in pre-mixed banana peel biochar with Fe_2_O_3_ after hydrothermal carbonization. Their results illustrated an increment of 407, 504, 451, and 446 m^2^ g^−1^ by combining with 0.05–1.0 g of the precursor solution, respectively. Similarly, Park et al. (2016) reported that during high pyrolysis temperatures (500–600 °C), the volume of pores in sesame straw increased from 0.0716 to 0.1433 cm^3^ g^−1^.

Furthermore, in another research, it is depicted that the surface area of *Saccharina japonica-*derived biochar was positively influenced by the temperature, resulting in a notable increase (2.9–175 m^2^ g^−1^), with the highest surface area achieved at 500 °C (X. Li et al. 2020a, b) noted that ZnCl_2_^−^ EBcan enhance the pore size up to 0.2–0.9 cm^3^ g^−1^. The BET (Brunauer, Emmett, and Teller) approach is frequently used to determine the SSA of biochar. In this method, the amount of N_2_ adsorbed on the surface of the biochar is evident at low temperatures (77 K) (Igalavithana et al. 2017). Apart from modification by nano-scale zero-valent iron and the effects of pyrolysis temperature, the subsequent impregnation of biochar with Mn or Mg enhances the surface area from 209.6 to 463.1, 12.68 to 174.29, and 244 m^2^ g^–1^, respectively (Deng et al. 2021; Hu et al. 2015; Liu et al. 2017).

On the contrary, several studies have documented a decrease in the SSA of functionalized biochar. The decline in surface area could potentially be attributed to the pore openings or pores being obstructed by chitosan (Burk et al. 2020). Recently, manganese oxide activation revealed a considerable decrease in SSA ranging from 60.97 to 3.18 m^2^ g^−1^ (Yu et al. 2017). Another research found that functionalized biochar through sulfur significantly affected surface area in contrast to pristine biochar. The highest surface area was observed in biochar generated at 500 °C and 700 °C (382 and 404 m^2^ g^−1^), whereas functionalization with sulfur led to a significant reduction in surface area to 10.06 m^2^ g^−1^ and 5.10 m^2^ g^−1^ (Jeon et al. 2020). Hence, it is crucial to thoroughly evaluate the characteristics of the final product concerning the impact of the modification process.

ii. Elemental Composition

Generally, the pristine biochar comprises 2–5% H, 45–60% C, and 10–20% O. Although the individual components employed to develop the product vary widely and rely on the feedstock. In order to assess the degree of hydrophobicity and carbonization in biochar, researchers commonly employed the O/C and H/C molar ratios (Kharel et al. 2019). Additionally, it includes minerals, such as Si, P, Ca, Al, and K, that are prominent inorganic components of biochar (Joseph et al. 2018). Many studies highlighted that type of feedstock, pyrolyzing procedures and functionalization may modify the properties of pristine biochar (Vijayaraghavan 2019). In one study, Mn-oxide-MB illustrated a surge in O (10.9%) and Mn (7.41%) concentrations. Meanwhile, similar research observed that C, H, and N levels declined from 85.3–73.0%, 1.75–0.33%, to 0.80–0.72% (Yu et al. 2017). This reduction was attributed to the activation process wherein previously MB was exposed to additional high temperatures, leading to the further breakdown of C, H, and N and their conversion into ash (Gai et al. 2014).

Furthermore, Wu et al. (2018) also reported a decline in H and C contents when biochar was magnetically modified with FeOS, Fe, and FeCl_3_. In FeOS biochar, C and H contents were the highest at C at 12.65% and 1.23%, while the lowest was in FeCl_3_-engineered at 9.16% and 0.28%, respectively. In addition to the mentioned modifications and their effects on the elemental constitution of biochar, various additional substances, such as chitosan and alkali/acid, have also demonstrated beneficial outcomes. Chitosan amendment resulted in a lower C percentage and increased H, O, and N ratios, confirming the inclusion of treated material on the surface of biochar (Hanbo et al. 2022).

iii. Alkalinity and pH

The pH of typical biochar usually varies from neutral to alkaline and is highly dependent on the type of feedstock, thermochemical process, and functional material, while studies about acidic biochar are also present (Jahirul et al. 2012; Qi et al. 2017). During the high pyrolysis temperature, the acidic functional groups (bionic acid) decompose, elevating the pH of biochar and causing an increase in inorganic alkali metal ions (Yuan et al. 2019). Additionally, several organic functional groups, including –COOH, –COO, –O, and –OH, can similarly raise the pH of biochar (Hongbo et al. 2017).

In another study, Zhou et al. (2013) recorded pH values of different biochars prepared from bamboo (7.9), sugarcane bagasse (7.5), hickory wood (8.4), and peanut husk (6.9), but after chitosan modifications, pH values changed to more alkaline 8.2, 8.1, 8.6, and 7.3, respectively. Similarly, the pH of corncob biochar produced at 600 °C was shown to be neutral (7.17), but after being modified by Mg-oxide, it reduced considerably to 10.4 (Ling et al. 2017). On the contrary, FeCl_2_-impregnated biochar exhibited an acidic characteristic (4.87) contrasted to conventional biochar (10.7) (Yin et al. 2017).

Moreover, hydrophilicity, hydrophobicity, sorption, and adsorption can be influenced by organic groups and linked with the buffering action of acid and base. Additionally, the organic groups present on the biochar surface carry negative charges, increasing the CEC (Munera-Echeverri et al. 2018). As the CEC of biochar increased, it ultimately surged in adsorption capacity (Hamid et al. 2020). Biochar modification by surface oxygenation via dry ozonization proved a promising technique for enhancing the CEC 10 times, in contrast to conventional biochar (Cheng et al. 2017). For instance, biochar derived from the limb of a pine tree and subjected to ozone gas for 1.5 h, the CEC of the biochar considerably elevated from 15.39 cmol kg^−1^ to 32.69 cmol kg^−1^. Although this technique decreased the pH and demonstrated the oxygenic functional group formation of the biochar surface (Huff et al. 2018).

iv. Functional Groups and Aromaticity

Several functional groups are associated with the surface of biochar, such as carboxylic, hydroxyl, and phenolic functional groups that contribute significantly to the remediation of contaminated soils (Gupta et al. 2016; Leng et al. 2020; Sajjadi et al. 2019). Among them, the most prevalent are O-containing functional groups, which can additionally be classified into neutral, and alkaline groups according to their inherent characteristics. The carboxyl, lactonic, and phenolic groups are examples of acidic groups, while the chromene and pyrone groups are known as basic active sites (Fig. 3). Their characteristics may be related to the carbon’s surface basic nature, which is more evident in carbon atoms without oxygen because of the existence of delocalized electrons (Tan et al. 2021).

The pyrolysis temperature significantly influences the functional groups present on the biochar surface. It is found that C=C and –CH_2_ functional groups could be successfully retained in pyrolysis, although C–O–C and –OH, C=O functional groups reduced with increasing pyrolysis temperature (Ying et al. 2019). Meanwhile, biochar’s water affinity, CEC, and polarity are regulated by oxygen-containing surface functional groups (Ying et al. 2019). Generally, the yield of biochar declines, while on the contrary, alkaline functional groups, pH, and ash concentration increase with increasing temperature (Yang et al. 2019). In a recent study, the adsorption capacity of MB derived from rice straw was observed to be higher than that of cotton straw-MB, likely due to the presence of additional functional groups (Rizwan et al. 2020).

The aromatic π-system is known to be involved in significant types of noncovalent specialized engagements termed electron donor–acceptor (EDA) interactions (Kah et al. 2017). The aromatic structures of biochar can significantly enhance pollutant adsorption, as they act as electron donors or acceptors and create bonds with the pollutants in soil (Dai et al. 2019). Usually, the carboxyl functional groups on the surface of carbonaceous adsorbents serve as electron acceptors, whereas the hydroxyl groups behave as electron donors (Ahmed et al. 2018). The aromaticity of biochar, determined using O/C and H/C ratios is highly influenced by pyrolysis temperature. The biochar derived from plant residues contains higher aromaticity due to high carbon material concentration. The cellulose and lignin decompose into small molecules that ultimately reduce H/C and O/C ratios as a result of depolymerization or dehydration. On the other hand, biochar from animal fecal and sludge does not possess any lignocellulosic molecules and hence avoids the depolymerization process (Zhang et al. 2018).

In a recent sulfamethoxazole structural analysis research, it was observed that the N-heteroaromatic ring, unprotonated sulfonamide group, and amino functional group act as significant *π*-electron acceptors, although they possess a high capacity to donate electrons (Tan et al. 2021). Similarly, Zhao and Zhou (2019) also reported *π*−*π* EDA interactions between biochar aromaticity and sulfamethoxazole. However, potential electron-donating groups identified include the phenolic −COOH−C−O and −OH groups (Chen & Carroll 2016; Na et al. 2017).

v. Hydrologic Properties

The ability of MB to resist water is a crucial quality that has a significant impact on soil water retention (Fig. 5). But the water retention property of biochar is highly dependent on the source of feedstock, synthesis technique, wetting characteristics, and particle size (Shaaban et al. 2013). The temperature during pyrolysis significantly influences the hydrologic characteristics when biochar is applied to soils (Kigozi et al. 2020). The most favorable hydrologic qualities were reportedly found in acid-functionalized biochar generated at higher temperatures (400–600 °C); in such amendment, water holding capacity was 18.45–22.45% higher than the control. This surge in water retention can be a result due to (i) high surface area and pore spaces and (ii) more hydrophilic functional groups that aid functionalized biochar (Głąb et al. 2016). Besides, Zn (23.85%) and Fe (22.45%), impregnated biochars also exhibited a considerable increase in water-holding capacity in soil against soil gravity losses —the mineral formation of Fe and Zn aids in water retention by increasing chemisorption and physisorption. For instance, hematite surfaces possess the capacity to sorb water 23–24 A^2^ per molecule, allowing it to adsorb the polar water molecules (Mosa et al. 2018). In contrast, the hydrologic properties of biochar are found to be independent of soil types, such as coarse-grained (non-cohesive) soil or fine-grained (cohesive) soil (Hussain et al. 2020). The mechanical resilience of aggregates at both micro and macro-scales was enhanced in biochar-amended soils, leading to notable improvements in cohesion and compressive behavior (Ajayi & Horn 2016; Hlaváčiková et al. 2019; Villagra-Mendoza & Horn 2018). There are possibilities that the hydraulic characteristics of soil that have been treated with EB extensively correlate with the quality and content of biochar, but these interactions are inadequately understood (Islam et al. 2021).

vi. Complexation and Van der Waals forces

Complexation is a special ability of biochar for adsorption that indulges ligand exchange, and bridging ligands are used to transport electrons (Mondal et al. 2020). The electron donors and acceptors engage to produce different compounds during the surface complexation. During the formation of these surface compounds, several polyatomic structures significantly improve the sorption potential of biochar (Haris et al. 2021). Besides, chelation is an exceptional kind of complexation that is described as an equilibrium process and distinguished by the development of a complex between organic molecules with more than one functional group (multiple bounds) and a single central atom (Barquilha and Braga 2021).

Several biochar features indicated that it may be an extremely effective adsorbent for the majority of soil contaminants and metals. Although these properties are highly influenced by the source of feedstock, pyrolysis time, and temperature, sufficient consideration should emphasize the analysis techniques of biochars produced under different circumstances.

The functionalized biochar contains a larger surface area and more functional groups than pristine biochar, such as carboxylic, phenolic, and lactonic functional groups that act as electron acceptors (Qiu et al. 2022) while the product of low pyrolysis possesses more electron-donating functional groups including amino –NH_2_ and hydroxyl –OH (Li et al. 2017; Y. Wang et al. 2019a, b). The complexion potential of biochar can improve if it is pyrolyzed in the presence of oxygen because of more surface oxidation. It is observed that plant-derived engineered biochars have higher complexion capacities for heavy metals in soils as compared to poultry litter and dairy manure-derived biochars (Ahmad et al. 2018; Ifthikar et al. 2018; Ji et al. 2022).

The van der Waals forces, also known as hydrophobic interactions, refer to intermolecular interactions that bind molecules together. These interactions are divided into two types: (i) weak London dispersion forces and (ii) stronger dipole–dipole forces (Ouyang et al. 2020). The van der Waals forces possess relatively modest energy about 0.4–4 kJ mol^−1^), while most of the sorbates are associated with biochar nonspecifically (weak London dispersion forces) (Rashidi and Yusup 2016). These forces specifically contribute to interactions between carbonaceous sorbents and ionic organic compounds because of the larger molecular size of IOCs as van der Waals forces increase with increasing contact area, and the high van der Waals coefficient of activated carbon (graphite) (Kah et al. 2017).

It is noted in a study that ZnCl_2_-MB enhanced the pore size up to 0.2 cm^3^ g^−1^ to 0.9 cm^3^ g^−1^ and the adsorption capacity of biochar via van der Waals forces (X. Li et al. 2020a, b). Similarly, montmorillonite-biochar composites pyrolyzed at 400 °C were also found to be highly effective for ammonium adsorption via Van der Waals interaction (Chen et al. 2017). Usually, the adsorption of organic pollutants on biochar occurs via weak physical adsorption without any strong chemical bonding. These weak bonds include hydrogen bonding, van der Waals forces, hydrophobic interactions, and electrostatic forces (Ahmed et al. 2018).

## Analytical techniques for modified biochar

The flexibility and interaction of biochar with soil are highly influenced by its various physical and chemical characteristics. Hence, a deep structural and physical analysis via appropriate methods has been proven beneficial (Fig. 3). If left unaddressed, the presence of a non-carbonized layer may lead to fluctuations caused by soil contaminants or other materials, potentially yielding inaccurate results (Ding et al. 2016; Panwar and Pawar 2020). A detailed summary of the physicochemical attributes of the MB employed in soil, along with a comprehensive overview of the advantages and disadvantages of the analytical techniques, is presented in Table 3.

**Table 3:** Summary of various analytical techniques of modified biochar

Technique	Utilization	Advantages	Limitation	Evaluated Biochars	Reference
Scanning Electron Microscopy (SEM)	• It is used to determine morphological differences between biochar surfaces and pores arrangement	• Limited need for conductive coatings due to high gas pressure • High magnification can be obtained in a range between 300000x and 500000x	• Not applicable for organic contaminants	• Magnetic biochar (γ-Fe_2_O_3_) • Magnetite (Fe_3_O_4_) biochar • MgO biochar nanocomposites	(Karunanayake et al. 2018; Mohammed & Abdullah 2018; Rong et al. 2019; Wei et al. 2021)
Energy Dispersive X-ray (EDX)	• It can evaluate the biochar surfaces and mapping of abundant elements	• Have the potential to precisely identify and assess the arrangement and existence of elements in the scanned domain • High-speed data collection and processing • Easy to handle	• Only detect the elements that have higher atomic numbers than boron • Highly dependent on probe current and voltage • Not applicable for organic contaminants	• S, Mn and Fe MB • Magnetic biochar (γ- Fe_2_O_3_)	(Abd Mutalib et al. 2017; Ballová et al. 2020; Lin et al. 2017; Rong et al. 2019; Scimeca et al. 2018; Yin et al. 2017)
Transmission Electron Microscopy (TEM)	• TEM is used to determine the surface morphologies like SEM • It can also analyze the lattice constant and chemical composition of biochar	• Very High magnification can be obtained in the range between 200000 and 1000000x • A multifunctional instrument that can be used for spectroscopy and nanoscale imaging	• It requires a high degree of vacuum to restrict the electron scattering they travel from the electron source to the electron optics	• Fe_3_O_4_ Biochar • Biochar nanoparticles (wheat straw)	(Chen et al. 2019, 2022; Tang & Yang 2017; Wang & Wang 2019)
X-ray Diffraction (XRD)	• It can analyze the composition of biochar, such as crystalline C or other materials • It is also used to determine organic compounds, including cellulose, hemicelluloses, and lignin, as well as inorganic compounds, such as oxides and sulfides carbonates	• A non-destructive technique that can produce 3D characterization of interface structure • It can be used with different combinations of in-situ methods • In-situ methods and Time resolution is possible	• Requires high-intensity X-ray beam • The usage is limited to access time to synchrotron source and single crystal surfaces	• S and Fe_3_O_4_-MB	(D. Chen et al. 2020a, b; Dutrow & Clark 2019; Gründer & Lucas 2016; J.-Y. Zhang et al. 2021a, b, c)
X-Ray Absorption Near Edge Structure (XANES)/ Near Edge X-Ray Absorption Fine Structure (NEXAFS)	• XANES/NEXAFS used to investigate the surface chemistry of highly complex types of C materials, for instance, charcoal • It can be used to identify the C species and stability with several structures at different pyrolysis time intervals (100–700 °C)	• Direct structural determination of any matter and isotope • Oxidation state and spin state direct determination	• Spectroscopy carried out in bulk gives an average structure • Very little information about the angle of the structure determines • Synchrotron X-ray source required	• Fe- EB • KOH steam–activated pecan shell biochar	(Igalavithana et al. 2017; Ippolito et al. 2012; Sutton et al. 2020; Wang & Feng 2021; J.-Y. Zhang et al. 2021a, b, c)
X-ray photoelectron spectroscopy (XPS)	• It is effective for surface characterization of biochar (surface elemental composition) • It can analyze chemical bonds, chemical, and the presence of distinct species of elucidated compounds on the surface of biochar	• It can carry sensitive surface quantitative analysis such as material composition and empirical formula (without hydrogen) • Have the potential to analyze the material up to the depth of 1–10 nm	• This technique is only applicable to solids since a high vacuum is needed • Highly time-consuming and cannot recognize hydrogen and helium atoms	• FeCl_3_, FeSO_4_, Fe/Ca, and Mn EB • MnO/NiO biochar composite	(Alqaheem & Alomair 2020; Chen et al. 2010; Ippolito et al. 2012; Lefebvre et al. 2019; Wang et al. 2016; Watts & Goacher 2022; Yoon et al. 2017)
Fourier‐transform infrared spectroscopy (FTIR)	• It can investigate mineralogy and chemical functional groups of biochar • Variation in the carbonation degree also makes it possible to determine	• The analysis can be performed on any matter (solid, liquid, or gas) • Fast and non-destructive data acquisition and processing • Mapping with a good special resolution of large surface samples is possible	• The interpretation of data is difficult, especially when working with a complex matter • Aqueous mixtures are complicated to investigate because water has a high infrared absorption capacity	• DOM/Cu MB • Fe/Mn EB	(Amen et al. 2020; Gniadek & Dąbrowska 2019; He et al. 2017, 2018; Lin et al. 2017; Talari et al. 2017)
Raman spectroscopy	• This technique is effective in quantifying the structural characteristics, especially graphite structures of biochar • Functional groups and crystalline C structures can be evaluated as well	• A precise single-point assessment carried out with an excellent special resolution • Spectra possess quantitative and qualitative information • It is feasible to acquire insight into the functional groups in the polymer	• Measurement parameters variation may affect the signal (e.g., laser wavelength) that negatively affects data interpretation	• Fe/Ca-EB • (NH_4_)_3_PO_4_ impregnated biochar • Magnetic biochar (γ-Fe_2_O_3_)	(Gniadek & Dąbrowska 2019; J. Li et al. 2020a, b; Silge et al. 2022; Yoon et al. 2017; C. Zhang et al. 2021a, b, c)
X-ray fluorescence spectroscopy (XRF)	• Commonly utilized to evaluate the compounds of biochar • Determination of inorganic compounds present on the surface of biochar • Very effective in quantifying the composition of biochar	• More powerful and precise for inorganic materials determination as compared to XRD	• Very costly	• Fe-EB • N and O activated	(Igalavithana et al. 2017; Jung et al. 2015; J. Li et al. 2020a, b)
Solid-state nuclear magnetic resonance (NMR)	• The structural composition of biochar and carbonization degree and stability can be determined by this method • It can be used to evaluate the contents of functional groups such as aromatic hydrocarbons, phenolic, methoxyl, and aliphatic in a biochar	• Findings for stability have strong correlations with several other techniques	• It gives deficient signals/noise ratio when encountering with high temperature pyrolyzed biochar • Signals obscured in the presence of ferromagnetic materials	• NaOH- MB • CO_2_ and steam-activated biochar • N and O activated biochars	(Guo et al. 2020; Igalavithana et al. 2017; Jung et al. 2015, 2013; Leng et al. 2019; Wakudkar & Jain 2022; Zhang et al. 2017)

## Effects of modified biochar on soil attributes

Modified biochar (MB) can alter soil functional groups, pore size, pore structure, surface area, and chemical properties. These modifications significantly impact soil quality by influencing its physical and hydraulic properties, nutrient profile, gas exchange characteristics, organic matter content, pH, electrical conductivity (EC), cation exchange capacity (CEC), and biological activities, including those of bacteria, fungi, and enzymes. The following provides a brief overview of how the addition of MB as a soil amendment affects soil quality.

i Soil pH

The presence of hydrogen (H^+^) and aluminum (Al^3+^) ions in the soil exchangeable sites causes acidity, severely affecting crop yield (Fig. 3). Pristine biochar is primarily alkaline in nature (Rashid et al. 2020). The pyrolysis temperature (> 400 ℃) produced biochar with alkaline pH (Novak et al. 2009). When applied to soil, organic compounds on the biochar surface will dissolve in water entering the pore spaces, increasing soil pH (Joseph et al. 2018). Also, biochar increases the sorption of nutrients and decreases acidity in acidic soils (Van Zwieten et al. 2012). However, the effectiveness of biochar in changing soil pH in acidic soils depends on the composition, properties of feedstock, and pyrolysis temperature (Fig. 4). Thus, several modifications of biochar have been developed. The activation of rice hull biochar by immersing with dimethyl dithiocarbamate sodium solution effectively decreased pH from 10.28 to 6.53 and improved soil properties (Wang et al. 2021). The application of Fe-MB in Cd-contaminated soils slightly increased pH units from 7.83 to 7.93 and helped to immobilize Cd in soil (Sun et al. 2021). In the case of red soils, the manganese-oxide-MB increased soil pH by 1.4 units while only 0.4 units increased in pristine biochar, thus preventing acidification in red soils (Yu et al. 2015). On the other hand, the pH of As-contaminated paddy soils (pH 7.12) were markedly decreased by Fe–Mn MB by around 0.75–1.16 units compared to the control (Lin et al. 2019a, b, c). Similarly, It was observed that soil resistance to acidity was enhanced through the application of HNO_3_/H_2_SO_4_-MB (He et al. 2022). Furthermore, thiourea-modified poplar-bark biochar was applied to Cd-contaminated soil, resulting in a 17% increase in soil pH as compared to the control, which led to an increase in base cations in the soil (Zhu et al. 2020). In multi-polluted soils, the magnetic biochar derived from eucalyptus wood and poultry litter increases soil-surged pH by 0.2–0.3 units (Lu et al. 2018). Likewise, the porous magnetic biochar from wheat straw was added to the soil and increased soil pH by approximately 6% (Fu et al. 2021). But in calcareous soils, steam-AB (acidic biochar) effectively decreases soil pH by 0.2–0.4 units (Ippolito et al. 2016), while in As-contaminated soil, Ca-MB causes an increase in pH (J. Wu et al. 2020a, b).

In contrast, rice straw MB with a 1:1 mixture of HNO_3_/H_2_SO_4_ and 15% H_2_O_2_ effectively improved pH buffering capacity and resistance to soil acidification compared to HCl-treated and unmodified biochar. The enhanced resistance was attributed to surface functional groups that increased soil resistance to acidification by generating protonation of organic anions, which retarded the decline in soil pH. However, HNO_3_/H_2_SO_4_ MB showed a higher number of carboxyl functional groups compared to the 15% H_2_O_2_-modified biochar, resulting in greater resistance to soil acidification. The application of HNO_3_/H_2_SO_4_ MB in paddy soil increased pH after wet-dry cycles, suggesting that this modification is an effective solution for remediating acidic soils. The underlying mechanism involves weak acid functional groups on the biochar surface that exist as organic anions in alkaline and neutral soils. Under acidic conditions, these anions protonate with H^+^ and convert to neutral molecules, inhibiting soil acidification and preventing a decline in soil pH (He et al. 2022; Shi et al. 2017, 2018),

Furthermore, Yu et al. (2015) found that soil pH increased with the application of 4% Mn oxide-MB. Similar results were observed with soil amendments using coconut shell and carrot pulp biochars modified by 8% thiourea (Gholami and Rahimi 2021; Liu et al. 2018). Other studies also showed increased soil pH with the application of 3% iron-zinc oxide composite-modified corn straw biochar (T. Yang et al. 2021a, b), 2% Fe-modified biochar (Moradi and Karimi 2021; Y.-Y. Wang et al. 2019a, b), 0.6% Brassica napus biochar-UV (Wang et al. 2021), and 0.6% Lolium perenne biochar-UV (Y. Zhang et al. 2021a, b, c).

ii. Soil moisture

Biochar application with a high surface area increases pore space and enhances soil moisture, which helps boost water retention capacity. Applying P-laden biochar considerably increased soil moisture by 93% over ordinary biochar when applied to acidic sandy soil planted with lettuce and improved seed germination and higher yield (Qincheng et al. 2018). Besides, soil moisture is enhanced by the EB derived from wood, switchgrass, and swine manure (Brassard et al. 2018). It is claimed that nano-biochar contains larger pores and increases the number of small pores. Thus, it could increase the soil moisture content more than pristine biochar (Yang et al. 2020). Nano-biochar application enhanced soil water infiltration and soil moisture content (Jinbang et al. 2016). Similarly, nano-biochar was found to decrease soil water loss due to high surface area (X. Chen et al. 2020a, b). Another, AB from the microwave catalytic process also increases water holding capacity by 98% more than ordinary biochar due to higher porosity, which shows a high correlation between WHC and micropore area (Mohamed et al. 2016).

In another study on composite-MB, particle-sized biochar, and acidified biochar, it was found that all these modifications enhanced the soil water-stable aggregate contents. Specifically, acid-modified biochar at the 0–15 cm soil layer increased soil–water aggregate content by 1.45–1.80 times compared to pristine biochar, while also enhancing soil moisture content and infiltration rate (Duan et al. 2021).

Similarly, An et al. studied biochar made from peach shells and pig manure, modified with H_3_PO_4_ and KOH, and applied in four dosages (0%, 2%, 3%, and 8%). They found that H_3_PO_4_-modified biochar had superior water retention compared to KOH-modified and pristine biochar, while KOH modification reduced the hydraulic functional groups on the biochar surface. Pig manure biochar demonstrated higher crack suppression intensity than that of other functionally activated biochars. However, the study generally recommended a 5–8% dosage for enhancing water retention and minimizing cracks (An et al. 2021).

iii. Dosage of application

The majority of biochar studies focused on application rates between 5 and 50 tons ha^−1^, resulting in increased crop yield and soil properties and remediating pollutants in soil (Das et al. 2020). However, this is not applicable in some cropping systems due to the high cost of production and transportation (Lehmann et al. 2021). However, several studies reported that biochar modification and application to the soil at certain rates effectively improve soil characteristics (Figs. 4, 5). Magnesium-oxide-derived biochar applied to saline rice paddy soils at 4.5 Mg ha^−1^ increases rice yields and adsorbs more phosphates (L. Wu et al. 2019a, b). Similarly, applying Mn-oxide-MB at an increasing rate (0.5%, 1%, and 2% wt/wt) surged rice biomass and decreased As concentration in the root and rice grains compared to conventional biochar (Yu et al. 2017). In a similar investigation, the application of 1% magnesium-impregnated biochar to soil (wt/wt) increased available P content in the surface soil by around 50% (Chen et al. 2018). On the other hand, Fe-MB applied to saline paddy soil at 4.5 Mg ha^−1^ increased P adsorption via co-precipitation of P to iron oxides, thus increasing P availability by 79–90% over control (L. Wu et al. 2020a, b). The yield of Cd and As-contaminated rice enhanced by Goethite-MB at the rate of (0.5%, 1.0%, and 1.5% wt/wt), the shoot biomass and root biomass increased around 56–88% and 55–98%, respectively. Also, goethite-MB decreases Cd and As concentration in rice tissues, improves photosynthesis capacity, and reduces oxidative stress (Irshad et al. 2020). Likewise, acidified biochar derived from various raw materials, including rice husk, sugarcane-bagasse, and wheat straw, applied in the soil at 1.5% wt/wt, increased plant nutrient uptake and biomass by around 40 and 30%, respectively (Qayyum et al. 2021). Nano-biochar applied to salt-affected soils at different rates (0, 0.10, 0.20, and 0.50% wt/wt) increased P adsorption capacity (Mahmoud et al. 2020). In another study, it was observed that nano-Fe-MB applied to soil at 0%, 0.05%, 0.1%, 0.2%, 0.4%, 0.8%, and 1.6% wt/wt concentrations reduced soil pH, Cd concentration and enhanced soil CEC. The application of lower rates (0.2–0.4%) of nano-Fe-MB effectively decreased Cd toxicity in the soil and rice. However, high nano-iron-MB application rates (0.8–1.6%) stimulated Cd toxicity in leaves. Thus, finding the optimum application rates should be taken into consideration to avoid risk and toxicity (Zhang et al. 2020). Nano-hydroxyapatite applied at different levels (1%, 5%, 10%, and 20%) in Pb-contaminated soils effectively immobilized Pb by around 44–57% after 28 days of incubation, effectively decreasing Pb toxicity in soils and accumulation in plants (Yang et al. 2016).

iv. The ratio of functional material saturation

The ratio of functional material to modify biochar was one of the primary gauges for the adsorption of pollutants (Wei et al. 2020). Biochar activation was done by soaking raw biochar in solution (30% H_2_O_2_, 1 M HCl, and 1 M KOH) (1:100 ratio) for 24 h to effectively adsorb Cr (VI). The ratio of Fe to nano-Fe-modified-biochar was 19.5%, effectively improving soil properties (CEC) and reducing Cd toxicity (Zhang et al. 2020). Similarly, Fe and Al-MB, at the ratio of 1 mol L^−1^ in FeCl_3_ and AlCl_3_, effectively increased soil pH from 6.93 to 7.30 and enhanced P retention capacity (Peng et al. 2021). Furthermore, magnetic biochar prepared by mixing biomass with a solution containing (a 1:1 molar ratio of FeCl_2_ and FeCl_3_) effectively remediates soil and adsorbed heavy metals, including As, Cd, and Pb (Wan et al. 2020). In a recent study, Mg–Al impregnated biochar at 1:20 g ml^−1^ (solid to liquid ratio) was observed to be adequate in adsorbing phosphate in the soil and contained a higher amount of oxygen functional groups (MgO and AlO) as compared to pristine biochar (Zheng et al. 2020). Similarly, MB nanoparticles exhibited a maximum adsorption capacity of 147 mg g^−1^, significantly higher than the 67.8 mg g^−1^ capacity of unmodified biochar (Wang & Wang 2018). This enhancement is attributed to the nanoparticles increasing the surface area and the number of functional groups on the MB surface. Moreover, KOH modification of biochar resulted in a substantial increase (approximately 2.4 times) in specific surface area and more than a 50% improvement in the adsorption capacity for Cd^2+^ and Cu^2+^ ions. Specifically, pristine biochar had a specific surface area of 189 m^2^g^−1^, whereas KOH-MB had a surface area of 455 m^2^g^−1^. The Cd^2+^ adsorption capacity of pristine biochar was recorded as 4.48 mg g^−1^, while KOH-treated biochar achieved a capacity of 6.81 mg g^−1^, representing a 50.7% significant increase. However, the Cu^2+^ adsorption capacity of unmodified biochar was 2.64 mg g^−1^, whereas the KOH-modified biochar showed a significantly enhanced capacity of 4.03 mg g^−1^ (Regmi et al. 2012).

v. Soil types

The physicochemical properties of soil, including texture, structure, pH, and organic matter content, can influence the interactions between MB and soil, consequently affecting the availability and mobility of nutrients and contaminants. (Yuan et al. 2019). It is worth noting that sandy soils with low CEC and organic matter content may have limited capacity for MB adsorption and nutrient retention. This, in turn, leads to an increased adsorption of MB and an improvement in nutrient retention (Liang et al. 2021). MB also increases the presence of organic matter. For instance, Kun et al. (2020) found a significant increase in soil C/N ratios and soil organic carbon after the application of Cd-binding biochar. Furthermore, (Yangyang et al. 2021) observed a higher accumulation of soil organic matter (SOM) and organic carbon with the application of rice husk biochar modified with NaOH, HNO_3_, and dimethyl dithiocarbamate sodium (3% w/w).

Similarly, Moradi and Karimi (2021) noted increased SOM and organic carbon with Fe-modified biochar (2% w/w). Higher organic matter was also observed with the application of 2% (w/w) Fe–Mn biochar (Wen et al. 2021), 1% (w/w) S-biochar (Y.-Y. Wang et al. 2019a, b), 1% (w/w) S-Fe biochar, 3% (w/w) (C. Wu et al. 2019a, b), iron-modified biochar, 8% (w/w) thiourea-modified biochar (Gholami & Rahimi 2021), 8% (w/w) carrot pulp biochar, and 3% (w/w) iron-zinc oxide composite modified corn straw biochar, compared to pristine biochar (T. Yang et al. 2021a, b). This variation can be attributed to the slower decomposition of biochar depending on the soil type. The formation of soil aggregates or organo-mineral complexes provides substantial physical protection to SOM, potentially preventing its decomposition. Additionally, the temperature sensitivity varying with soil type limits short-term decomposer access to SOM, while the majority of decomposition is carried out by slower microbial metabolism (Rittl et al. 2020). For instance, in a study by Feng (2010), it was observed that the temperature sensitivities of biochar vary in soils with different textures and mineralogy. The temperature coefficient (*Q*10) values of biochars ranged from 1.93 to 2.20 in Oxisols and from 2.74 to 2.77 in Vertisols, within a temperature range of 20–40 °C. Additionally, the presence of biochar was found to alter the *Q*10 values of native SOM as well, depending on the soil type (Fang et al. 2017, 2014).

## Future prospective

Following the advancements and findings outlined in earlier sections, future research on MB should prioritize several key areas to address existing gaps and enhance our understanding. These research efforts can be categorized into four main segments. However, to achieve optimum results, collaborative efforts among experts from different domains are strongly recommended.

- ***Analytical advancement***
- **Developing Advanced Techniques**: Employ more sophisticated techniques such as Pyrolysis Gas Chromatography-Mass Spectrometry (Py-GC–MS) and Thermogravimetric-Fourier Transform Infrared Spectroscopy-Mass Spectrometry (TG-FTIR-MS) to elucidate biochar formation mechanisms more accurately.
- **Advanced Spectroscopy**: Apply advanced spectroscopic methods to gain deeper insights into the functionalization of MB, allowing for better control over functional groups and porosity.
- **Integrated Approaches**: Use a combination of unique spectroscopic investigations and theoretical modeling to enhance the understanding of factors impacting biochar efficiency after soil amendment.
- ***Future perspectives in fundamental and material research***
- **Mechanism Investigation**: Explore the mechanisms behind changes in biochar properties due to different pyrolysis conditions or modification procedures. Prioritize sustainable feedstock materials and their functionalization for specific contaminants to promote a green and sustainable environment.
- **Synergistic Integration**: Investigate the potential for synergistic integration with other treatment modalities, such as combining MB with biofilters, to enhance biochar's adsorption capabilities and address environmental challenges.

### Emerging environmental research concerns

- **Regeneration Processes**: As biochar research advances, it brings forth new challenges and dilemmas. A primary concern revolves around regeneration processes. Addressing how to efficiently desorb pollutants adsorbed on biochar for subsequent safe treatment and optimizing the recycling of the adsorbed biochar stands as paramount issues requiring meticulous consideration.
- **Environmental Risks**: A critical aspect under scrutiny is the risk of secondary pollution arising from MB. For instance, there is a pertinent question regarding the possible interaction of metal-MB with other environmental compounds following its application in soil and leaking into groundwater. Understanding the possibility of antagonistic effects with different compounds and developing effective methods for separating treated biochar from soil and groundwater are pressing issues that demand immediate attention in future research endeavors.
- ***Future research in aging and stability***
- **Long-Term Effects**: Investigating the long-term effects of MB on soil functions and its behavior across diverse soil types necessitates immediate attention. A recent study illustrated the direct influence of MB on soil biological communities' composition and abundance, as well as affects the retention of applied pesticides (Wang et al. 2022). Consequently, weed control in MB-amended soils may pose challenges, as the efficacy of preemergence herbicides could diminish.
- **Environmental Behavior:** MB proved to facilitate the formation of soil aggregates, enhance the physical protection of organic carbon, and increase yield production, with other positive environmental impacts such as the decrease in methane and gross greenhouse gas emissions. However, these initial positive effects might shift to negative over time, necessitating attention to the long-term environmental behavior of MB. It is particularly crucial to investigate how biochar interacts with minerals and how such interactions affect the performance of soil and water remediation over short-term and long-term aging. Addressing these issues in future research is imperative for a comprehensive understanding of MB's role and impact on soil.
- **Simulated Aging Methods**: There is a need to develop simulated aging methods that integrate multiple artificial aging processes. Specifically, considering the aging time scale in natural environments, it is crucial to quantitatively explore response models of MB properties using various artificial aging methods. This approach will enhance our understanding of how biochar evolves over time under environmental conditions and improve the accuracy of predictions regarding its behavior and efficacy in practical soil applications and its implications for different crop systems.
- **Geochemical Behavior**: In the context of pollutant management by MB, there exists a pressing need for further exploration into the geochemical behavior of emerging contaminants under long-term application MB. Understanding how MB aging interacts with and influences the fate of emerging contaminants across diverse environmental contexts is crucial for devising effective pollutant remediation and management strategies. For instance, investigating potential scenarios such as the movement of MB to groundwater and the release of metals in the case of metal modification. However, the impacts of such interactions are contingent upon soil types, with sandy soils often exhibiting more significant benefits compared to others (Kavitha et al. 2018). As a result, there is an urgent need for extensive research on the long-term effects of MB across various soil types.

In summary, focusing on these future research areas will advance our understanding of MB and its applications. An integrated and collaborative approach will be essential for achieving comprehensive and impactful results.

## Conclusion

In conclusion, this article explores the physicochemical characteristics of biochar altered through various techniques, emphasizing the significance of comprehending the impact of these alterations on biochar properties. This insight will assist readers in discerning effective methods to modify biochar for specific applications. Our assessment suggests that MBs have significant potential for improving soil quality and health, and mitigating greenhouse gases. Furthermore, environmentally-friendly modifications can enhance energy and carbon storage in soils. Additionally, this review will offer comprehensive insights into advanced analytical techniques developed to analyze modifications necessary prior to application, enabling precise targeting of specific objectives. However, further investigation is required to analyze the relationships between material composition, structure, and energy storage. Overall, this article contributes to our understanding of the potential of MB as a versatile and sustainable material with significant environmental and economic benefits.

## Outlook

This comprehensive review thoroughly explores the recent advances in utilizing Modified biochar (MB), specifically in soils. Beyond mere synthesis, it delves into the evolved physicochemical characteristics of MB, providing invaluable insights into its efficacy and limitations. Indeed, comprehending the unique properties endowed by each modifying agent is paramount, as they directly shape the characteristics of the final product. Therefore, it is imperative to fully grasp the attributes of both MB and the modifying agent prior to production, enabling targeted applications within soil environments.

However, in the rapidly advancing world, it is essential to ascertain the exact nature and properties of processed MB. Hence, our review underscores the significance of embracing advanced analytical methodologies to characterize modified biochar and gauge its effectiveness precisely. By doing so, we aim to shed light on the crucial role these techniques play in ensuring biochar quality and performance in modern applications. Furthermore, this paper contains a comprehensive discussion of the mechanisms behind the factors influencing the efficiency of MB post-soil application.

Additionally, we have highlighted the need for future research to explore the mechanisms behind alterations in biochar properties due to different modifications. Our future research perspective section is delineated into four segments. Firstly, analytical advancement advocates for leveraging sophisticated techniques, such as Pyrolysis Gas Chromatography-Mass Spectrometry (Py-GC–MS) and Thermogravimetric-Fourier Transform Infrared Spectroscopy-Mass Spectrometry (TG-FTIR-MS) to deepen our comprehension of biochar formation mechanisms. Additionally, it underscores the significance of advanced spectroscopic methods in elucidating modified biochar functionalization for precise control over functional groups and porosity. Secondly, future research in fundamental and material research underscores the importance of synergistically integrating MB with other treatment modalities, and investigating the mechanisms underlying changes in biochar properties resulting from modification procedures.

Moreover, concerns regarding risks associated with MB are pointed out in the environmental research section, such as the risk of secondary pollution by potential leakage into groundwater after application, the possibility of antagonistic effects, and the development of new regeneration processes to enhance pollutant absorption efficiency. Lastly, the roadmap for aging and stability future research delves into the necessity of focusing on the long-term effects of MB on soil functions and its behavior across diverse soil types. This entails comprehensively understanding MB role and impact on soil by developing simulated aging methods that integrate multiple artificial aging processes. This will enhance the accuracy of predictions concerning MB's behavior and efficacy in practical soil applications and its implications for different crop systems.

## Data Availability

The datasets used or analyzed during the current study are available from the corresponding author upon reasonable request.
